# Delusional Ideation, Cognitive Processes and Crime Based Reasoning

**DOI:** 10.5964/ejop.v13i3.1181

**Published:** 2017-08-31

**Authors:** Dean J. Wilkinson, Laura S. Caulfield

**Affiliations:** a Department of Psychology, University of Worcester, Worcester, United Kingdom; b Bath Spa University, Bath, United Kingdom; Webster University Geneva, Geneva, Switzerland; University of Liverpool, Liverpool, United Kingdom

**Keywords:** delusional ideation, crime based reasoning, cognition

## Abstract

Probabilistic reasoning biases have been widely associated with levels of delusional belief ideation (Galbraith, Manktelow, & Morris, 2010; Lincoln, Ziegler, Mehl, & Rief, 2010; Speechley, Whitman, & Woodward, 2010; White & Mansell, 2009), however, little research has focused on biases occurring during every day reasoning (Galbraith, Manktelow, & Morris, 2011), and moral and crime based reasoning (Wilkinson, Caulfield, & Jones, 2014; Wilkinson, Jones, & Caulfield, 2011). 235 participants were recruited across four experiments exploring crime based reasoning through different modalities and dual processing tasks. Study one explored delusional ideation when completing a visually presented crime based reasoning task. Study two explored the same task in an auditory presentation. Study three utilised a dual task paradigm to explore modality and executive functioning. Study four extended this paradigm to the auditory modality. The results indicated that modality and delusional ideation have a significant effect on individuals reasoning about violent and non-violent crime (p < .05), which could have implication for the presentation of evidence in applied setting such as the courtroom.

Individuals engage in reasoning processes as they interact and exist within the world (Green & Gilhooly, 2005). Whilst people have the ability to successfully navigate their way through everyday complex situations, ‘biases’ or errors in reasoning have been detected whilst individuals solve simple reasoning problems in a psychological laboratory setting (Verschueren, Schaeken, & d’Ydewalle, 2005). This setting potentially differs to everyday life in that the environment is controlled and usually the individual is focussing on a single task, as opposed to everyday life where individuals are processing multiple streams of rich sensory information (Wilkinson, Caulfield, & Jones, 2014; Wilkinson, Jones, & Caulfield, 2011).

## Delusional Ideation in Clinical Populations

It is argued that reasoning impairments contribute to the formation and maintenance of delusional beliefs (Coltheart, Langdon, & McKay, 2011; Connors & Halligan, 2015). An influential study being Huq, Garety, and Hemsley (1988) of the beads task paradigm. ‘Probabilistic style’ reasoning (Oaksford & Chater, 2001), plays a central role in the conditional inference process (Oaksford, Chater, & Larkin, 2000), and can lead to errors in individuals reasoning (Evans, Ellis, & Newstead, 1996; George, 1997; Stevenson & Over, 1995). Probabilistic reasoning bias’ have been widely reported in clinical populations with a diagnosis of a psychosis (Garety, Hemsley, & Wessely, 1991; Hemsley & Garety, 1986; Huq, Garety, & Hemsley, 1988) which suggests that under conditions of uncertainty, patients with delusions demonstrate a `jumping to conclusions' (JTC) style of reasoning, requiring less information to come to a decision, and being more confident about the decision that they have reached (e.g. Dudley, John, Young, & Over, 1997; Fear & Healy, 1997; Garety et al., 1991; Huq et al., 1988). However, recent studies, such as Ross et al. (2016), proposes that it is analytic cognitive style – defined as the willingness or disposition to critically evaluate outputs from intuitive processing and engage in effortful analytic processing – that predicts data gathering on a bead task rather than delusional ideation.

## Delusional Ideation in Healthy Populations

The continuity approach has gathered a wealth of support with regards to considering delusions and other features of psychosis being measurable on a continuum that extends from and includes clinical and nonclinical population (Freeman, Pugh, Vorontsova, Antley, & Slater, 2010; Galbraith, Morgan, Jones, Ormerod, Galbraith, & Manktelow, 2014; van Os, Linscott, Myin-Germeys, Delespaul, & Krabbendam, 2009). Schizotypy is a sub-clinical category of experience which captures individuals who present schizo-psychopathological characteristics but are not extreme enough to be classified as requiring clinical attention of diagnostic (Claridge & Beech, 1995). Gruzelier (1996) suggests that schizotypy consists mainly of impulsive non-conformity, social anxiety, positive features such as unusual perceptions, and negative features such as introversion.

Researchers, such as Galbraith, Manktelow, and Morris (2008, 2010), have conducted a number of studies exploring psychopathological tendency primarily composed of samples of non-pathological individuals. Galbraith et al.’s approach, using a psychometric test to screen for schizotypal tendencies, circumvent issues of medication effects, motivation and the nature and severity of the symptoms and experiences over time faced when testing a clinical sample (Galbraith, Manktelow, & Morris, 2010; Thurston et al., 2008). Furthermore, moral and ethical responsibilities of testing clinical patients are overcome (Galbraith, Manktelow, & Morris, 2010).

## Crime Based Reasoning

Decision making in the real world includes reasoning about crime based scenarios (Wilkinson, Caulfield, & Jones, 2014; Wilkinson, Jones, & Caulfield, 2011). A wealth of research has argued that decision making processes in forensic and legal setting are subject to errors and biases (Alesina & La Ferrara, 2014; Gunn et al., 2016; Sonnemans & van Dijk, 2012). Sonnemans and van Dijk (2012) reviewed the types of errors investigated and documented including judicial decision errors and biases. Scientific investigations and methods of research do not easily lend themselves to exploring the judicial system and therefore other modes and methods of representative environments, such as mock scenarios and mock court rooms, have been utilised (Carter & Mazzula, 2013; Kapardis & Farrington, 2015; Krauss & Lieberman, 2016).

Probabilistic style decision making is applied by a jury to criminal cases during court proceedings, with the outcome being to assess the likelihood of a guilty or not guilty verdict based on the evidence provided (Sonnemans & van Dijk, 2012). However, probabilities are not always assessed, in this context, quantitatively and different legal systems present information in differing styles, amounts and timings (Sonnemans & van Dijk, 2012). The rate of decision making, including the amount of information gathered before making a decision could have profound affects in practice, for example, jury decision making (Nicholson, Yarbrough, & Penrod, 2014). Previous research has considered the relationship between schizotypy and crime based reasoning using qualitative methods (Wilkinson, Caulfield, & Jones, 2014; Wilkinson, Jones, & Caulfield, 2011) and found clear qualitative self-reported differences in probabilistic reasoning styles from their participants.

This study aimed to assess the relationship between delusional beliefs and probabilistic style reasoning of crime based scenarios using a series of experiments that manipulated the presentation of evidence through auditory and visual modalities whilst also introducing a distraction utilising a dual taking paradigm, mimicking the type of processing that may happen in an applied setting such as a courtroom. It was hypothesised that individuals with high scores for delusional beliefs, compared to low scorers, would require fewer pieces of information before making a decision. It was also hypothesised that the distraction task would increase the difference observed between high and low scorers in terms of information gathering.

## Experiment One

### Method

#### Participants

Through convenience sampling, forty-five (11 male and 34 female) student volunteers from various undergraduate courses at a west-midlands UK based university took part in this study. The age of the participants ranged between 19 and 44 (*M* = 24.33, *SD* = 6.82). No other demographic information was collected.

#### Design

This study adopted a mixed (one within and one between factor) 2x2 experimental design. The study consisted of a within participant factor, violent (emotionally arousing) and non-violent scenarios, and a between participant factor, high and low scorers on the *Peters Delusions Inventory* (PDI). The dependent variable was data gathering (which was a measure based on a scale to rate how much information an individual required before making a decision).

#### Materials and Procedure

The following measures were presented to participants. The *Peters Delusions Inventory* (Peters et al., 1999) – paper form- is a 21-item measure of delusional ideation. The scale has good levels of reliability and validity (Peters et al., 2004). The response format is a 5 point scale for distress, preoccupation, and conviction in relation to the 21-items presented. The *Computerised Visual Reasoning Task* (CVRT) was specially designed to measure individuals crime based decision-making about whether the character in two scenarios ‘had done the right thing’. Participants were able to gather as much or as little information as they desired about a given scenario before making a decision. This concept derived from traditional reasoning tasks, such as the beads tasks, except applied to a manufactured but realistic life scenarios.

The violent scenario (emotionally arousing) used a story about a character that attacked a ‘youth’ in the street (see Appendix 1). The attack was based on a number of assumptions. Following the short story about the sequence of events leading to the attack were subsequent statements that provided additional information and described a more complete picture of the events. Each additional statement was displayed on screen for as long as the participant wished to view. The non-violent scenario was based on a story about a character that lied in order to borrow money from a man with no intention to pay him back. The statements following the short story described vital information with regards to exposing the truth behind the character’s need for the money. Participants were required to indicate at which point they were happy to make a decision by pressing the D key and the number of required statements recorded. 

### Results

#### Descriptive Statistics

The reasoning task results were analysed using SPSS statistics 17.0 and are presented below. Descriptive statistics for the ‘data gathering’ scores can be viewed in Table 1. The descriptive statistics suggested that high PDI scorers require less information compared to low PDI scorers for both violent and non violent crime scenarios, although this effect is represented to a greater extent in the non violent crime scenario.

**Table 1:** Data Gathering Descriptive Statistics

Crime Type	Low PDI (*n* = 15)		High PDI (*n* = 15)		*df*	*F*	*p*
	*M*	*SD*	*M*	*SD*			
Violent					1,43	0.16	.69
	2.57	2.53	2.14	2.25			
Non violent					1,43	6.96	.04
	3.71	2.34	1.79	1.42			

Inferential Statistics. A two-way mixed ANOVA suggested that there was not a significant interaction between PDI and Crime Type, *F*(1, 41) = 3.15, *p* = .08. Further analysis showed a low effect size (*d* = 0.18) according to Cohen’s *d* (Cohen, 1992).

#### Non-Violent Scenario and ‘Data Gathering’

Whilst a non-significant interaction was found overall, a significant effect was highlighted when analysing PDI on ‘data gathering’ with regards to reasoning about non-violent scenarios, *F*(1, 41) = 6.96, *p* = .01. Further analysis revealed a large effect size (*d* = 1.02) according to Cohen’s *d* (Cohen, 1992).

### Discussion

The data collected from the reasoning task suggest that there were no significant differences between high and low PDI scorers, when measuring the amount of information required before making a decision about a violent crime scenario. However, there was a significant difference when reasoning about a non-violent scenario. Despite the non significant results from the violent scenario, it is still possible to see a trend in the mean ‘data gathering’ scores that suggests that high scorers requested less information when reasoning about a violent crime. It seems that low scoring individuals required more information, when compared to high scoring individuals, before making a decision or coming to a conclusion. The implications of this could be detrimental in setting such as the court room where in an adversarial process a jury is making a decision based on information that is staggered in presentation.

## Experiment Two

Little research has explored the impact that modality (auditory vs. visual) may have on reasoning biases, particularly when considering delusion ideation and especially given the characteristics, such as deficits in auditory sensory “echoic” memory (Umbricht et al., 2000) associated with ‘schizotype’ experiences. These deficits lead to difficulties in extracting relevant information from sensory stimuli across all modalities (Javitt et al., 2000).

As such, individuals who experience deficits in extracting relevant information may produce biases on tasks that require the utilisation of represented extracted information. In other words, some individuals are overwhelmed with the mass of information available through their senses, and are unable to filter out the relevant or important information (Delhommeau, Dubal, Collet, & Jouvent, 2003). This study, therefore, adapted the materials from experiment one to present in an auditory format to consider the affect that modality has on crime based reasoning when considering delusional ideation. It was hypothesised that the auditory version of the experiment would capture greater differentiation between high and low scorers on delusional ideation in terms of the amount of information required before they made a decision.

### Method

#### Participants

Fifty-five university students participated in this study. All participants were undergraduate students from a range of faculties and degree courses across the University. Participants were aged between 19 and 52 (*M* = 23.8, *SD* = 8.01), ten were males and forty-five females.

#### Design

A 2x2 experimental design was adopted for this study. Similar to study one, independent variable one was based on PDI scores and independent variables two was based on scenario type (violence and non-violent).

#### Materials and Procedure

Consistent with study one, the 21-item *Peters Delusions Inventory* (Peters et al., 1999) was used to measure delusional ideation (see study one for more information). The *Computerised Auditory Reasoning Task* (CART) was specially designed for this study, which was an adaptation from study one. The auditory reasoning task presented the same information as study one but through an auditory modality, given the evidence of cross modality bias occurring in individuals with schizotypy as well as a small amount of evidence for differentiation in psychosis prone individuals (Ferstl, Hanewinkel, & Krag, 1994; Reed et al., 2008). Once again, Eprime programming software was used to program, present and capture participant’s responses. Participants wore a head set in order to listen to the crime based scenarios and additional information. Given that the information was delivered to participants through auditory presentation, statements could be heard once unlike study one where participants could read and re-read on screen.

## Results

Descriptive statistics. The reasoning task results were analysed using SPSS statistical analysis software 17.0, and are presented below. Descriptive statistics for the ‘data gathering’ scores can be viewed in Table 2.

**Table 2:** Data Gathering Descriptive Statistics

Crime Type	Low PDI (*n* = 19)		High PDI (*n* = 19)		*df*	*F*	*p*
	*M*	*SD*	*M*	*SD*			
Violent					1,53	23.18	.02
	6.78	3.95	2.22	3.04			
Non violent					1,53	8.82	.01
	6.06	2.65	3.11	3.27			

### 

#### Inferential Statistics

The results from the Levene’s pre-test were non significant and therefore did not violate any assumptions for parametric testing (Levene’s p > 0.05). A two-way mixed Analysis of Variance (ANOVA) was performed on the results of the reasoning task, therefore considering the independent variables of PDI (high and low) and crime type (violent and non-violent), and the dependent variable data gathering. The two-way mixed ANOVA revealed a non-significant interaction between PDI and scenario type, *F*(2, 32) = 15.04, *p* < .001. Further analysis showed a large effect size according to Cohen’s *d* (*d* = 1.3), and retrospective power = 0.99 (Cohen, 1992).

### Discussion

The results from this study demonstrated, through a two-way analysis of variance (ANOVA), that overall there was no interaction between PDI and crime type, however, the *p* and *F* values suggested significant differences between high and low scorers within each crime type (violent and non violent). Either individuals who scored high for Schizotypal tendencies required fewer ‘chunks’ of information before making a decision (data gathering), compared to individuals who scored low for Schizotypal tendencies, or it is possible that low scorers gathered more information in comparison to high scorers. The descriptive statistics suggest that the violent crime scenario, which was potentially more emotionally arousing, created a bigger gap between the mean ‘data gathering’ scores generated by the high and low scoring groups. Therefore, it could be argued that the violent crime scenario enhanced the ‘jump to conclusions’ bias that frequently occurs in individuals at risk of delusions (Huq, Garety, & Hemsley, 1988) or with high levels of delusional ideation (Galbraith, Manktelow, & Morris, 2010), or caused low scorers to gather further information before making a decision. Furthermore, the results suggest that the biases in reasoning that accompany delusional beliefs, which have presented themselves on traditional non-specific reasoning tasks, also present themselves when making decisions about crime based situations which could have implications in legal settings when undergoing probabilistic decisions based on case evidence (Sonnemans & van Dijk, 2012).

## Experiment Three

### Dual Processing Theory

Dual process theories have provided an alternative explanation to previous single system theories which propose that cognitive processes such as reasoning are governed by a single system (Braine, 1990; Johnson-Laird, 1983; Rips, 1994). Dual process theories, therefore, arguably stand in contrast to modular models of human cognition (Barrett & Kurzban, 2006; Carruthers, 2006; Cosmides & Tooby, 1992; Sperber, 1994). Dual processing accounts of reasoning and human behaviour have been developed by both cognitive and social psychologists (Manktelow, 2012), the relevance of which is the theoretical application to ‘higher’ cognitive processes which include thinking, reasoning, decision making, and social judgment (Evans, 2008). All dual process theories share the common idea that there are two differing modes of processing: System One and System Two (Kahneman & Frederick, 2002; Stanovich, 1999). The first system, occasionally referred to as the heuristic system (De Neys, 2006), solves problems based on an individual’s prior knowledge and beliefs. The second system, sometimes referred to as the analytic system, allows reasoning according to logical standards, which requires access to a central working memory system of limited capacity. As a result, System One is assumed to operate rapidly and automatically, whereas the operations of the analytic system are believed to be slow and heavily demanding of resources (De Neys, 2006). These two systems can act in concert and consequently the heuristic system will usually provide a fast, frugal and correct conclusion. However, heuristic processing can lead to biased reasoning in situations that require more elaborate and analytic processing. This occurrence leads to conflict between the two systems (Stanovich & West, 2000).

Study three explored whether the results of study two were indicative of modality, visual vs. auditory, or whether auditory processing requires a dual process system. It was hypothesised that an additional active task would increase the differentiation of reasoning scores for high and low scorers.

### Method

#### Participants

74 participants from a west midlands University took part in this study. The participants were undergraduate students from a range of Faculties and degree courses across the University. Participants were aged between 18 and 54 (*M* = 22.5, *SD* = 6.69), 23 were males and 51 females. It was ensured during the recruitment stage that all participants were first language native English speakers.

#### Design

The 2x2x2 experiment designed enabled the exploration of three independent variables: PDI (between factor determined by the scores on the Peters Delusions Inventory: Peters & Garety, 1996); scenario type (within factor representing non-violent and violent); and memory task (within factor compiled of high and low memory load) explored using a dot matrix memory task. There was one dependent variable which was the amount of information required before making a decision based on a 0-8 scale (data gathering).

#### Materials and Procedure

Consistent with previous studies presented in this paper the *21-item Peters Delusions Inventory (**Peters et al., 1999**)* was used to measure delusional belief ideation (see previous studies for more information). The *Dual Processing Visual Computerised Decision Task (DPVCDT)* was specially developed for this study. This task was an adaptation of the reasoning task used in experiment one to present information to participants in a visual modality. Statements were present on screen for participants to observe for as long as they wished. In addition to previous studies in this paper, the dot matrix memory task (dual task) was completed. Both of these tasks were presented and completed using E-Prime stimulus software. This design explores whether ‘dual tasking’ as opposed to a change in modality, and therefore greater demands on processing, enhances the effects of biases in individuals who score high for delusional beliefs. This is supported by the evidence of bias’ occurring in individuals with Schizophrenia, schizo-type disorders, as well as a small amount of evidence for differentiation in psychosis prone individuals (Ferstl, Hanewinkel, & Krag, 1994; Reed et al., 2008).

### Results

#### Descriptive Statistics

The reasoning task results were analysed using SPSS statistics analysis software 17.0, and are presented below. Descriptive statistics for the ‘data gathering’ scores can be viewed in Table 3.

**Table 3:** Data Gathering’ Descriptive Statistics

Crime Type	Low PDI (*n* = 25)		High PDI (*n* = 25)		*df*	*F*	*p*
	*M*	*SD*	*M*	*SD*			
Violent (hard)					1,48	11.84	< .001
	4.72	4.03	3.04	3.05			
Violent (easy)					1,48	18.31	< .001
	5.2	3.99	2.68	2.69			
Non Violent (hard)					1,48	2.52	.12
	4.32	3.53	2.88	2.83			
Non Violent (easy)					1,48	2.99	.09
	5.68	3.65	3.48	3.16			

#### Inferential Statistics: ‘Data Gathering’

The data gathering results were analysed using a 3 way mixed Analysis of Variance (ANOVA) to assess the impact of one between subject independent variables (PDI: high and low) and two within subject independent variables (Scenario type: violent and non-violent; Memory load: high and low) on participants ‘data gathering’ scores.

Box’s test of equality of covariance matrices was significant (*p* = .04) and therefore the results below are reported using the Greenhouse-Geisser correction.

There was no significant interaction between memory and PDI, *F*(1, 48) = 2.69, *p =* .11, η^2^ = 0.05, PDI and Scenario type, *F*(1, 48) = 0.12, *p =* .73, η^2^ = 0.00, memory and scenario, *F*(1,48) = 1.18, *p =* .28, η^2^ = 0.02, and memory, scenario and PDI, *F*(1, 48) = 0.00, *p* = .96, η^2^ = 0.00).

There was a significant difference in mean data gathering between high and low scorers, *F*(1, 48) = 6.79, *p* = .01. There was also a significant main effect of memory (easy / hard: *p* = .03) but there was no significant main effect of scenario type (*p* = .65).

### Discussion

The data presented no significant interactions, in any combination, between PDI, memory load and crime type. This could be interpreted, explained and accounted for in a number of ways. It is possible that the experiment design is not sensitive enough to capture any relationships between PDI, dual systems of processing and crime scenario type, despite adopting tools and methods that had been used previously in a number of studies which had generated significant results (De Neys, 2006; Evans, 2008; Galbraith et al., 2010).

The results suggest that the biases that occurred in experiment one and two were not a result of overloaded resources and increased demands placed on memory but rather the impact of modality (visually or auditory processed information). Nonetheless, it is impossible to be conclusive without testing the dual process paradigm within the auditory modality.

It is also possible that dual process theory does not adequately account for aspects of crime based real world reasoning and hence there is no relationship or interaction between the two separate systems when reasoning about crime based scenarios. It is also possible that the two systems of processing do not impact upon one another when individuals are engaged with crime based reasoning. This would suggest that decisions can be made in the presence of other cognitive tasks, therefore, what is important is the modality of presented information in relation to delusional ideation. The implications of this study would suggest that in an applied setting, such as a courtroom, it is the mode and delivery of information that is important for decision making rather than the number of processes occurring in the setting.

## Experiment Four

Experiment four was designed as an adaptation of study three but delivered and presented in an auditory format. It was hypothesised that the auditory format would enhance and elevate the effects of a reasoning bias.

### Method

#### Participants

Sixty-One participants took part in the auditory Dual Processing study. The participants were recruited from a west midlands University. The sample consisted of undergraduate students from a range of faculties and degree courses across the University. Participants were aged between 18 and 38 (*M* = 22.8, *SD* = 5.41), 21 were males and 40 females.

#### Design

A 2x2x2 experimental design was adopted for this study. Independent variables PDI (high and low), crime type (Violent and non-violent) and memory task (High and Low). The dependent variable was data gathering (the amount of information participants required to make a decision).

#### Materials and Procedure

The study comprised three main component measures. As with the previous studies in this paper, the *21-item Peters Delusions Inventory* (Peters et al., 1999) was used to measure delusional belief ideation (see study one for more information). The *Dual Processing Auditory Computerised Decision Task (DPACDT)* was developed especially for this study. The task was based on the visual crime based reasoning task in experiment three, however, the renovated design presented the scenarios and statements to participants in an auditory modality accompanied by a visual dot matrix memory task. The tasks were presented and results were recorded using E-Prime stimulus software. This design explored whether reasoning biases are further enhanced by ‘dual tasking’ or whether the modality of presented information impacts upon individuals decisions (Ferstl, Hanewinkel, & Krag, 1994; Reed et al., 2008).

Participants were presented with either a simple or difficult dot matrix memory test which they were required to remember whilst reading a crime based scenario accompanied by additional statements. Participants were required to indicate at which point they were happy to make a decision about whether the character in the story had done the right thing. Participant’s responses were recorded on a ten-part scale. Once participants had completed the crime scenario, they were then requested to recall the dot matrix memory task. This process was repeated to account for violent and non violent as well as simple and difficult conditions.

### Results

#### Descriptive Statistics

The reasoning task results were analysed using SPSS statistics analysis software 17.0, and are presented below. Descriptive statistics for the ‘data gathering’ scores can be viewed in Table 4.

**Table 4:** Data Gathering Descriptive Statistics

Crime Type	Low PDI (*n* = 20)		High PDI (*n* = 20)		*df*	*F*	*p*
	*M*	*SD*	*M*	*SD*			
Violent (hard)					1,46	10.37	< .001
	6.50	3.08	2.08	1.99			
Violent (easy)					1,46	11.39	< .001
	6.88	3.17	2.33	1.69			
Non Violent (hard)					1,46	0.52	.48
	6.21	2.96	2.55	2.46			
Non Violent (easy)					1,46	14.87	< .001
	6.54	3.27	2.41	1.77			

#### Inferential Statistics: ‘Data Gathering’

The data gathering results were analysed using a 2x2x2 mixed Analysis of Variance (ANOVA) to assess the impact of one between subject independent variables (PDI: high and low) and two within subject independent variables (Scenario type: violent and non-violent; Memory load: high and low) on participants ‘data gathering’ scores.

Box’s test of equality of covariance matrices was significant (*p* = .00) and therefore the results below are reported using the Greenhouse-Geisser correction.

There was no significant interaction between memory and PDI, *F*(1, 46) = 0.48, *p =* .49, η^2^ = 0.01, PDI and Scenario type, *F*(1, 46) = 0.95, *p* = .33, η^2^ = 0.02, memory and scenario, *F*(1, 46) = 0.57 *p* = .81 η^2^ = 0.00, and memory, scenario and PDI, *F*(1, 46) = 0.04, *p* = .85, η^2^ = 0.00.

There was a significant difference in mean data gathering when comparing high and low PDI scorers, *F*(1, 46) = 70.7, *p* < .001.

### Discussion

As with experiment three, the analysis of the data gathering results found no significant relationships between PDI, dual processing and crime type, suggesting that these factors do not impact upon one another. However, there were significant differences highlighted between high and low PDI scorers with regards to their data gathering scores consistent with previous findings. High PDI scorers required fewer pieces of information before coming to a conclusion in comparison to low scorers who require more pieces of information before making a decision. However, the memory tasks did not interfere with this finding and caused no further elevated signs of reasoning biases. This suggests that the dual processing (Evans, 2008) account does not provide an explanation for why biases are elevated when presented in a visual modality.

## Discussion and Conclusion

The results gathered from the series of experiments, overall, are consistent with previous studies of delusional ideation and a ‘jump to conclusions’ bias (Freeman, Pugh, Vorontsova, Antley, & Slater, 2010; Galbraith, Morgan, Jones, Ormerod, Galbraith, & Manktelow, 2014; van Os, Linscott, Myin-Germeys, Delespaul, & Krabbendam, 2009) and therefore a ‘jump to conclusions’ bias can be applicable to crime based reasoning as well as everyday scenarios. Although it appears that memory load does not affect the relationship between delusional ideation and a ‘jump to conclusions’ bias, other variables do appear to enhance the affect.

Experiment one and two produced particularly interesting findings with regard to both reasoning biases and influential factors surrounding the intensity of those biases. It was concluded from experiment two that delusional ideation and crime based reasoning related to either modality, visual or auditory presented information (Delhommeau, Dubal, Collet, & Jouvent, 2003), or the increase load on memory resources which naturally occur when remembering information that has been received through the auditory senses. However, when examining the shortfalls of experiment one, it was clear that the methodological design adopted for this study made it impossible to identify whether the causal factor was modality or indeed competition for working memory resources. Therefore, experiment three and four provided a solution to address this problem by adopting a dual task design (Evans, 2008). This allowed for an investigation of whether an increase in memory load enhances the crime based reasoning biases identified by experiment one and two. The outcome of these additional studies suggest that it is not increased load on memory and resources that enhances the biases and therefore it can be deduced that there are key differences when reasoning about crime using verbally presented information compared to visually presented information. The results reported in experiment two demonstrated that individuals with Schizotypal tendencies required fewer ‘pieces’ of information before making a decision, compared to individuals who scored low for Schizotypal tendencies. There was a significant difference in both non-violent and violent crime scenarios with regard to individuals; ‘data gathering’ scores. However, the violent crime scenario created a bigger gap between the mean ‘data gathering’ scores generated by the high and low scoring groups. Therefore, it could be suggested that the violent crime scenario enhances the ‘jump to conclusions’ bias that frequently occurs in individuals at risk of delusions (Huq, Garety, & Hemsley, 1988). Furthermore, the results suggested that the biases in reasoning that accompany delusional ideation, have presented themselves on traditional non-specific reasoning tasks, also present themselves on crime based reasoning tasks given the right conditions.

The findings from the series of experiments could have implications, or at least pose questions, in applied areas such as the court room. It seems that levels of delusional ideation relate to the amount of information individuals require to make a decision about crime based scenario which is a process of decision making that occurs in court cases, particularly in cases and systems that include a jury. Consequently, future studies might explore this relationship in a mock court room setting, or individuals making decisions in the presence of other individuals and other individuals responses.

## Appendix 1

### Violent Scenario

Jared was walking home from work late one night when he heard a scream from the road ahead. He ran down the road to find a lady lying on the floor calling for help. She told Jared that she had been attacked by a gang. As there were lots of people surrounding the lady by this point, Jared ran further down the road in the direction that the lady had said the gang had gone. Jared caught up with a group of lads who were running down the road. He shouted at them and managed to capture one of them by the hood. Losing his temper he threw the guy to the floor and punched him.

Did Jared do the right thing?  The young man that Jared attacked ended up in hospital

The lad that Jared had assaulted had nothing to do with the attack on the lady

The lady had been causing trouble in the neighbourhood

One of the gang members was an ex-boyfriend of the lady

The police were monitoring the gang and all confrontations should have been reported to the police

Jared had previously confronted neighbours about noise levels and they had threatened to hurt his fiancé

The attack towards the lady had left her with a broken arm, sprained wrist and black eye

The lady Jared had found in the street was his fiancé

That was the final statement, did Jared do the right thing?
